# A new *Atrococcus* species (Hemiptera, Coccomorpha, Pseudococcidae) from China, with a key to Chinese species

**DOI:** 10.3897/zookeys.950.49300

**Published:** 2020-07-20

**Authors:** Jiang-Tao Zhang, Jia-Ying Zhou, You-Liang Pan, Xing-Ping Liu

**Affiliations:** 1 College of Forestry, Jiangxi Agricultural University, Nanchang 330045, China Jiangxi Agricultural University Nanchang China; 2 Key Laboratory of National Forestry and Grassland Administration for the Protection and Restoration of Forest Ecosystem in Poyang Lake Basin, Nanchang 330045, China National Forestry and Grassland Administration for the Protection and Restoration of Forest Ecosystem in Poyang Lake Basin Nanchang China

**Keywords:** *Atrococcus rushuiensis*, Jiangxi, mealybug, new combination, *Sporobolus fertilis*, taxonomy

## Abstract

A new mealybug species *Atrococcus rushuiensis* Zhang, **sp. nov.**, collected under the leaf sheath of *Sporobolus fertilis* (Poaceae) in Fuzhou City, Jiangxi Province, China, is described and illustrated. A new combination is introduced, transferring *Allotrionymus shanxiensis* Wu to the genus *Atrococcus* as *A. shanxiensis* (Wu), **comb. nov.** A key is presented for the species of *Atrococcus* recorded from China.

## Introduction

The genus *Atrococcus* Goux, 1941 (Pseudococcidae, Pseudococcinae) was established with *Atrococcus melanovirens* Goux as its type species. With oral rim ducts present, *Atrococcus* is morphologically similar to *Allotrionymus* Takahashi, *Chorizococcus* McKenzie, *Spilococcus* Ferris, and *Vryburgia* De Lotto (Wu 2000). Due to the inadequate definition, different authors have applied different combinations of characters for generic separation (e.g., Williams 1962; Tang 1992; Danzig and Gavrilov-Zimin 2015). Based on a study of mealybugs from Russia and neighbouring countries, Danzig and Gavrilov-Zimin (2015) found that there is no difference between *Allotrionymus* and *Atrococcus*, and treated *Allotrionymus* as a junior synonym of *Atrococcus*. Hence, we follow Danzig and Gavrilov-Zimin (2015) in regarding *Allotrionymus* as a junior synonym of *Atrococcus*.

In China, Tang and Li (1988) first recorded *Atrococcus* from Inner Mongolia and reported three species: *A. achilleae* (Kiritchenko) (on the root of *Bassia scoparia*), *A. innermongolicus* Tang in Tang and Li (on the root of *Artemisia apiacea*) and *A. paludinus* (Green) (on *Leppula intermedia*). Later, Tang (1992) recorded two *Allotrionymus* species: *Al. elongatus* Takahashi (on *Heteropappus altaicus*) and *Al. multipori* Kawai (on *Chloris radiata*) from Inner Mongolia; the former was a misidentification of *Chorizococcus scorzonerae* Tang found by Wu (2000). Meanwhile, Tang (1992) also transferred *Spilococcus pacificus* (Borchsenius) to *Atrococcus* and *Trionymus plurostiolatus* Borchsenius to *Allotrionymus*. The above-mentioned four species were placed under *Atrococcus* by Danzig and Gavrilov-Zimin (2015). Subsequently, Wu (1999) described a new species *Allotrionymus calamagrostis* Wu (under the leaf sheath of *Calamagrostis* sp.) from Henan; this species was placed in the genus *Atrococcus* by Danzig and Gavrilov-Zimin (2015). Recently, Wu (2000) provided a study of *Atrococcus* and its related genera, reporting a new species, *Allotrionymus shanxiensis* Wu (under the leaf sheath of *Melica scabrosa*) from Shanxi, which is transferred to *Atrococcus* as *A. shanxiensis* comb. nov. following the present generic diagnosis. Wu (2000) also reported a new Chinese record, *A. cracens* Williams, collected from Inner Mongolia (on *Artemisia halodendron*?) and Shanxi (on *Artemisia* sp. and *Heteropappus altaicus*). Including the new species described here, there are ten *Atrococcus* species recorded in China: *A. achilleae*, *A. calamagrostis*, *A. cracens*, *A. innermongolicus*, *A. multipori*, *A. pacificus*, *A. paludinus*, *A. plurostiolatus*, *A. rushuiensis* sp. nov., and *A. shanxiensis* comb. nov.

In this study, a new species, *A. rushuiensis* Zhang, sp. nov., is described from China, and a key to the Chinese *Atrococcus* species is also provided.

## Materials and methods

All mealybug specimens were collected from under the leaf sheaths and transferred into 75% alcohol, then prepared and mounted mainly according to the method of Borchsenius (1950). The terminology for the morphological features used in the description are mainly explained by Williams (1962, 2004). Photograph was taken with a Nikon D7500 camera. The descriptions and measurements were made using a light microscope (SOPTOP BH200) fitted with an ocular micrometre, and six slide-mounted specimens were studied for measurements. Measurements are in micrometres (μm) except the lengths and widths of the bodies are given in millimetres (mm); all measurements are given as minimum and maximum. Drawings are presented as is usual for Coccomorpha, with the central drawing showing the outline of the body and the distribution of characters (dorsum on the left side, venter on the right), with the enlarged details (not to scale) showing the structure of important characters around the margin.

All specimens examined are deposited in the College of Forestry, Jiangxi Agricultural University, Jiangxi, China.

## Taxonomy

### Atrococcus

Taxon classificationAnimaliaHemipteraPseudococcidae

Goux, 1941

0B1DF47F-245D-50BA-8B0D-A22A7624DA63

Pseudococcus (Atrococcus) Goux, 1941: 69. Type species: Atrococcus melanovirens Goux by original designation.

#### Generic diagnosis.

Body in life usually greenish or white, females of some species show black coloration after being placed in ethanol or potash. Body of adult female on slide oval to elongate oval. Antennae seven- or eight- segmented. Circulus present or absent. Legs well developed, claw without a denticle. Both pairs of ostioles well developed. Anal ring usually situated at apex of abdomen, bearing six setae. Anal lobes moderately developed, each bearing a normal apical seta. Cerarii numbering 1–17 pairs, each cerarius usually bearing two conical setae. Trilocular pores numerous, evenly scattered on all body surface. Multilocular disc pores usually present on venter, more rarely present on dorsum. Oral rim ducts present, forming transverse rows on dorsum and sometimes present on venter. Oral collar tubular ducts present, at least on venter, sometimes present on dorsum. Most species have group of oral collar tubular ducts on prothorax in front of anterior spiracles, often accompanied by a group of multilocular disc pores. Flagellate setae of different sizes present on both body surfaces (adapted from Tang 1992, Danzig and Gavrilov-Zimin 2015).

### Atrococcus rushuiensis

Taxon classificationAnimaliaHemipteraPseudococcidae

Zhang sp. nov.

478FC5C8-6209-50E5-8AEA-F3809D2E3734

http://zoobank.org/FB9C2A23-2BA9-4646-879B-0423B1B92B6B

Figures 1 , 2

#### Material studied.

***Holotype*.** ♀ (mounted singly on a slide), China, Jiangxi Province, Fuzhou City, Rushui Forest Park [27°58'N, 116°22'E], under the leaf sheath of *Sporobolus fertilis* (Poaceae), 2.x.2019, coll. Jiang-Tao Zhang. ***Paratypes*.** 8 ♀♀ (mounted on 8 slides), same data as holotype.

#### Etymology.

The species name is based on the collection locality, Rushui Forest Park.

#### Description.

**Figure 1.**
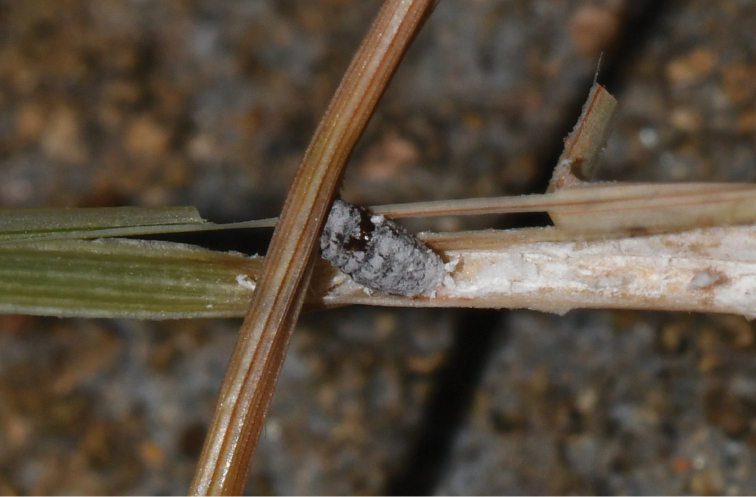
Habitus photograph of *Atrococcus rushuiensis* sp. nov. under the leaf sheath of *Sporobolus fertilis* (Poaceae).

Alive: body elongate, dark reddish, with thin covering of white mealy wax, and only caudal filaments present (Fig. 1).

**Figure 2.**
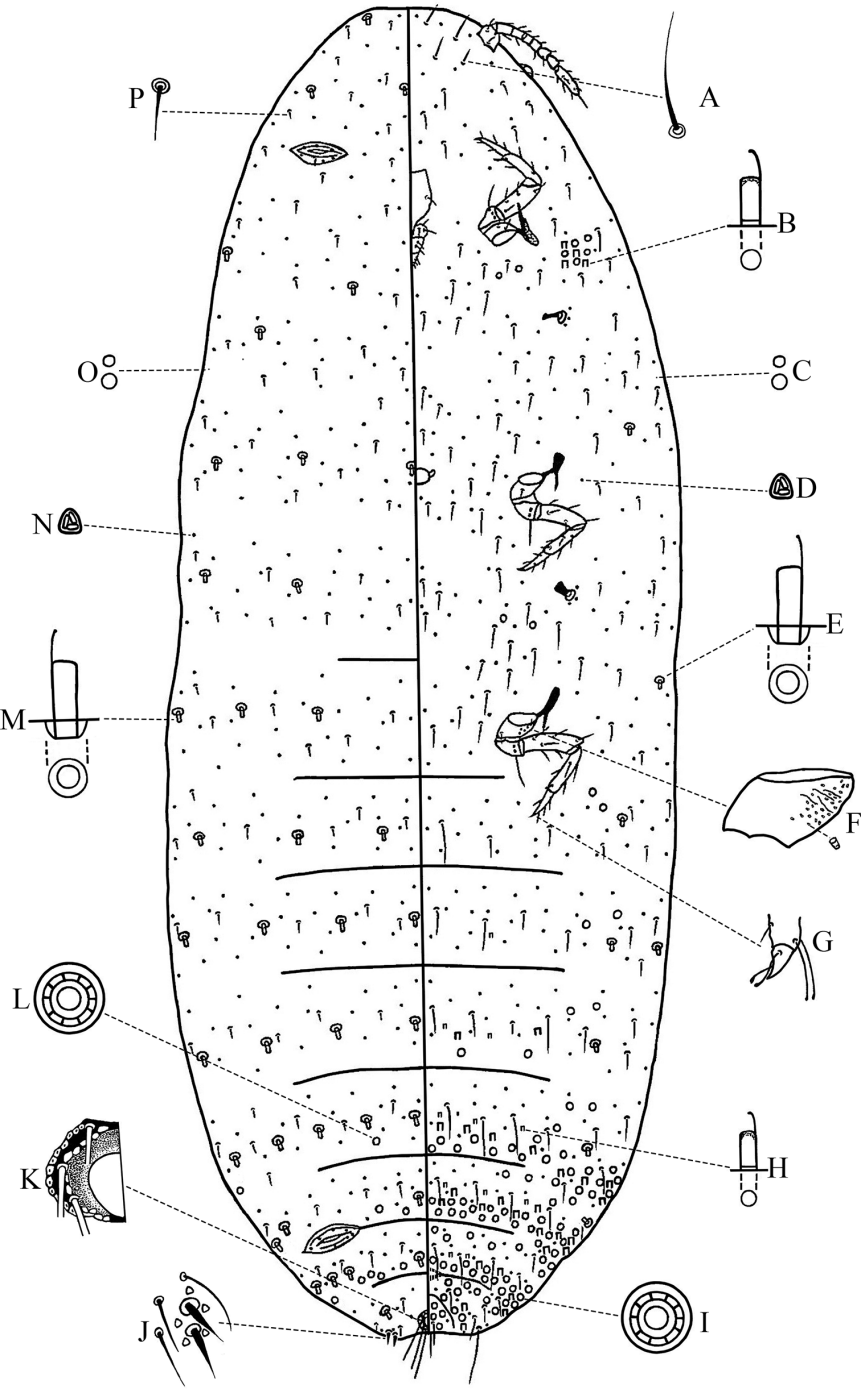
Adult female of *Atrococcus rushuiensis* sp. nov. Venter (**A–I**) **A** flagellate seta **B** large type of oral collar tubular duct **C** disc pore **D** trilocular pore **E** oral rim duct **F** hind coxa **G** claw **H** small type of oral collar tubular duct **I** multilocular disc pore. Dorsum (**J–P**) **J** anal lobe cerarius **K** anal ring **L** multilocular disc pore **M** oral rim duct **N** trilocular pore **O** disc pore **P** dorsal seta.

Slide-mounted specimens (*N* = 6): body of adult female (Fig. 2) elongate, 2.5–3.1 mm long, 0.9–1.4 mm wide. Anal lobes moderately developed, ventral surface of each lobe bearing an apical seta, each 100–122.5 μm long. Antennae eight-segmented, 267.5–289 μm long, lengths of each segment: I 42.5–52.5, II 37.5–38.8, III 23.8–27.5, IV 18.8–22.5, V 17.5–23.8, VI 20–26.3, VII 28.8–32.5 and VIII 65–76.3 μm. Eye spot located at body margin lateral to antennal base. Legs well developed; hind coxa 52.5–65 μm long, hind trochanter + femur 185–215 μm long, hind tibia + tarsus 198.8–240 μm long; claw 17.5–22.5 μm long, both tarsal digitules and claw digitules knobbed, longer than claw. Ratio of lengths of hind tibia + tarsus to hind trochanter + femur 1.07–1.14:1. Ratio of lengths of hind tibia to tarsus 1.49–1.69:1. Translucent pores present, minute duct-like, present on anterior and posterior surface of hind coxa. Circulus absent. Clypeolabral shield 125–145 μm long. Labium with three segments, 62.5–70 μm long. Ostioles moderately developed, each lip with 4–13 trilocular pores and 0–2 short setae. Anal ring normal, 61.3–70 μm in diameter, bearing six long setae, each seta 82.5–103.8 μm long. Cerarii numbering a single pair on anal lobes only. Anal lobe cerarii (C_18_) each containing two slender conical setae, each seta 18–21 μm long, with 2–3 auxiliary setae, and 4–5 trilocular pores near conical setae base, all situated on a membranous area.

***Dorsum*.** Setae short and slender, each 15–25 μm long. Trilocular pores each 3.5 μm in diameter, evenly distributed. Oral rim ducts each 9–10 μm long, 6 μm wide, in more or less single transverse rows on most segments. Oral collar tubular ducts absent or present, if present, each 6–7 μm long, 3 μm wide, having fewer numbers marginally on abdominal segments VI or VII. Multilocular disc pores each 7–8 μm in diameter, forming transverse rows or scattered on medial abdominal segments V–VII (V has 0–7 pores, VI has 1–7 pores, VII has 3–12 pores), occasionally few present on margin of abdominal segments V–VII. Discoidal pores minute, scattered.

***Venter*.** Setae slender, longer than those on dorsum, each 36.3–80 μm long. Trilocular pores similar to those on dorsum, evenly distributed. Oral rim ducts same as those on dorsum, present on margin and submargin areas of thoracic and abdominal segments. Oral collar tubular ducts of two types: a large type, similar to those on dorsum, present in transverse rows across abdominal segments III–VIII or IV–VIII, also in marginal groups on abdominal segments V–VIII or VI–VIII, and a small group (together with multilocular disc pores) present on prothorax in front of anterior spiracles (4–11 ducts and 6–17 pores); a small type, each 5 μm long, 2 μm wide, mainly distributed across middle areas of abdominal segments III–VIII or IV–VIII, a few also present on margin with large ducts. Multilocular disc pores same as those on dorsum, numerous, present posterior to vulva, in transverse rows at posterior edges of abdominal segments IV–VII, in transverse rows at anterior edges of abdominal segments VI–VII, a few occurring on submargin areas of abdominal segments II–IV near oral rim ducts, also forming groups along margin of abdominal segments V–VIII or VI–VIII. Discoidal pores minute, scattered.

#### Host plant.

Poaceae: *Sporobolus fertilis*.

#### Distribution.

China: Jiangxi (Fuzhou).

#### Biology.

Living under the leaf sheath of its host plant.

#### Remarks.

*Atrococcus rushuiensis* sp. nov. is very similar to *A. luffi* (Newstead) in the number of cerarii and multilocular disc pores present on both body sides, but it differs from the latter by the following features (condition of *A. luffi* given in parenthesis): (i) dorsal margin oral collar tubular ducts absent or few (numerous, with multilocular disc pores in submarginal groups up to segment III); (ii) ventral oral rim ducts absent in median areas of prothorax and mesothorax (present in these areas); (iii) translucent pores duct-like (normal, not duct-like) [The morphology of *A. luffi* is mainly based on Williams (1962)].

The new species also resembles *A. paludinus* in possessing fewer than 20 oral rim ducts on each segment, which is different from *A. luffi* in having about 20 oral rim ducts on each segment, but differs from the latter by the following features (condition of *A. paludinus* given in parentheses): (i) cerarii numbering one pair only (cerarii numbering 6–7 pairs); (ii) Translucent pores duct-like (normal, not duct-like) [The morphology of *A. paludinus* is also mainly based on Williams (1962)].

In *A. rushuiensis* sp. nov., the number of ducts and pores vary among individuals, which belong to intraspecific variation. Some specimens have only a small number of ducts and pores, but in other specimens those ducts and pores are much more numerous.

### Key to adult females of *Atrococcus* known from China

**Table:** 

1	Multilocular disc pores present on venter and dorsum	**2**
–	Multilocular disc pores present on venter only	**5**
2	Dorsal tubular ducts absent or present in compact groups along abdominal margin only	**3**
–	Dorsal tubular ducts present and forming transverse rows	***A. achilleae* (Kiritchenko)**
3	Cerarii present in 3–7 pairs	**4**
–	Only one pair of cerarii present	***A. rushuiensis* sp. nov.**
4	Cerarii 6–7 pairs, dorsal multilocular disc pores on margins only of posterior abdominal segments	***A. paludinus* (Green)**
–	Cerarii 3–4 pairs, noticeable groups of multilocular disc pores in submedian areas of dorsum	***A. cracens* Williams**
5	Circuli present	**6**
–	Circuli absent	***A. innermongolicus* Tang in Tang and Li**
6	Oral rim duct present on venter and dorsum	**7**
–	Oral rim duct present on dorsum only	**8**
7	Circuli 2–5 in number	***A. plurostiolatus* (Borchsenius)**
–	One circulus present	***A. multipori* (Kawai)**
8	Prothoracic group of tubular ducts absent	**9**
–	Prothoracic group of tubular ducts present	***A. pacificus* (Borchsenius)**
9	Translucent pores normal, also extend to metathorax cuticle near hind coxae	***A. calamagrostis* (Wu)**
–	Translucent pores duct-like, only present on the cuticle of hind coxae	***A. shanxiensis* (Wu) comb. nov.**

## Supplementary Material

XML Treatment for Atrococcus

XML Treatment for Atrococcus rushuiensis
